# Detection of Apple Hammerhead Viroid, Apple Luteovirus 1 and Citrus Concave Gum-Associated Virus in Apple Propagation Materials and Orchards in the Czech Republic and Hungary

**DOI:** 10.3390/v14112347

**Published:** 2022-10-25

**Authors:** Eva Várallyay, Jaroslava Přibylová, Zsuzsanna Nagyne Galbacs, Almash Jahan, Tunde Varga, Josef Špak, Ondřej Lenz, Jana Fránová, Jiří Sedlák, Igor Koloniuk

**Affiliations:** 1 Genomics Research Group, Department of Plant Pathology, Institute of Plant Protection, Hungarian University of Agriculture and Life Sciences, Szent-Gyorgyi Albert Street 4, 2100 Gödöllő, Hungary; 2 Czech Academy of Sciences, Biology Centre, Institute of Plant Molecular Biology, Branišovská 31, 37005 České Budějovice, Czech Republic; 3 Research and Breeding Institute of Pomology Holovousy, Ltd., Holovousy 129, 50801 Holovousy, Czech Republic

**Keywords:** apple, virus, viroid, rootstock, aphid, transmission, HTS

## Abstract

Grafting cultivars onto rootstocks is a widely used practice by the apple industry predominantly aimed at faster fruit bearing. Using high-throughput sequencing, we revealed the presence of recently described viral agents, namely apple hammerhead viroid (AHVd), apple luteovirus 1 (ALV-1), and citrus concave gum-associated virus (CCGaV), in germplasm collections and production orchards in the Czech Republic and Hungary. The HTS results were validated with RT-(q)PCR, and Northern blotting techniques. To obtain further insight about the presence of these agents, RT-PCR based surveys were carried out and showed their widespread presence alone or in mixed infections. The pathogens were present both in production areas and in feral samples. In addition, rootstock-to-scion transmission of ALV-1 and CCGaV was confirmed using commercial rootstock materials. Phylogenetic relationships based on partial sequences of distinct variants were also investigated. Furthermore, the rosy apple aphid was found to be ALV-1-positive, suggesting that it might be a potential vector of the virus.

## 1. Introduction

Apple trees (*Malus domestica*) are affected by several pathogens [1,2]. Among these, viruses have been of special interest for many years, starting with viral agents causing apple mosaic disease in the 1950s [3]. Although there are many viral and viroid species known to infect apples, only five agents (apple chlorotic leafspot virus—ACLSV, apple mosaic virus—AMV, apple stem grooving virus—ASGV, apple stem pitting virus—ASPV, and apple scar skin viroid) are tested during the propagation of apple materials [1,4].

Rootstocks are propagated in a vegetative, clonal manner on an industrial scale. This might promote the dissemination of latent infections of any non-screened pathogens, including viruses or viroids. Such latent infections can later develop into acute form and/or increase the severity of diseases caused by other pathogens during mixed infection, abiotic stress, or susceptibility of the grafted varieties.

For fast and efficient screening of propagating material it is essential to know a spectrum of potential pathogens. High-throughput sequencing (HTS) facilitated the discovery of various novel viral species, overcoming the technical limitations of traditional plant virology methods (e.g., transmission to indicator plants or purification of viral particles) [5,6,7]. Importantly, the concentration of certain apple viruses is rather low, which directly impairs their detection and diagnostics [1]. At the same time, HTS practically reversed the traditional direction of plant pathology studies (starting with disease and moving towards potential causative agents), but many recently discovered intracellular pathogens are not yet linked to diseased phenotypes [6,8,9].

The apple hammerhead viroid (AHVd; family *Avsunviroidae*, genus *Pelamoviroid*) was initially discovered by a prediction computational algorithm with HTS data of apple small RNAs [10]. Later, the prediction was validated by RT-PCR and Northern blotting. Its autonomous replication was further evidenced by infection using in vitro transcripts. Modelling of its secondary structure revealed a hammerhead conformation containing stabilizing kissing loop interactions [11]. The original studies did not report any specific symptoms in the AHVd-positive trees [10,11]. Further AHVd discoveries in symptomatic trees showed the presence of common apple viruses [12,13], making any correlation between viroid infection and the observed symptoms impossible. AHVd was subsequently reported worldwide in apple trees in Africa [14], North America [13], Japan, New Zealand and Europe [13,15,16], as well as in one non-apple species, loquat [17].

In contrast to the pathway of AHVd discovery, apple luteovirus 1 (ALV-1; family *Tombusviridae*, genus *Luteovirus*) was revealed by HTS of apple trees affected by rapid apple decline (RAD) disease [18]. The disease develops gradually and starts with leaf discolouration and trunk cracking, and tree vigour is severely affected, eventually resulting in rapid decline. In addition to North America, ALV-1 was reported in Korea [19], Greece [20] and recently in Belgium [16].

Citrus concave gum-associated virus (CCGaV; order *Bunyavirales*, family *Phenuiviridae*, order *Phlebovirus*) was previously discovered in a citrus and was shown to be a causative agent of citrus concave gum-blind pocket disease, a disease known for decades with unknown aetiology [21]. The diseased trees had deeply depressed trunk concavities and leaf chlorotic flecking symptoms. However, not all CCGaV-positive *Citrus* samples were symptomatic. The virus was then identified in the second host, an apple, in the United States (from a French imported material), China, Brazil, and Italy [18,22,23,24,25].

In this study, we investigated the occurrence of AHVd, ALV-1, and CCGaV in germplasm, commercial rootstocks, production orchards, and feral trees in the Czech Republic and Hungary. The data were complemented by experimental virus graft transmission assays and screening for potential aphid vectors.

## 2. Materials and Methods

### 2.1. Plant Material and RNA Extraction

A total of 316 apple leaf samples were taken from production orchards and germplasm collections in the Czech Republic and Hungary, together with samples from feral/private orchards and imported rootstock material in the Czech Republic (Table A1, Table S1). The Czech leaf samples were processed with a GeneJET Plant RNA Purification Kit (Thermo Fisher, Waltham, MA, USA), and the extracted RNA was quantified and quality-controlled using a NanoDrop ND-1000 (Thermo Fisher). The Hungarian leaf samples collected from four different branches of the trees were used for RNA extraction by the CTAB method [26].

### 2.2. sRNA Sequencing Library Preparation

For sRNA HTS, the RNA pools representing individual trees or a mixture of ten different trees (in the case of the 8_Apple_biotest library) were prepared by mixing equal amounts of RNA (Table A1). The sRNA sequencing libraries were prepared from the purified small RNAs using a TruSeq Small RNA Library Preparation Kit (Illumina, San Diego, CA, USA) and our in-house modified protocol [27]. In total, 16 small RNA libraries were prepared and sequenced using a single index on a HiScanSQ by UD-Genomed (Debrecen, Hungary) (100-bp, single-end sequencing). FASTQ files of the sequenced libraries have been deposited in GEO (GSE205183).

### 2.3. Bioinformatic Evaluation of sRNA HTS

We used the CLC Genomics Workbench (Qiagen, Hilden, Germany) for bioinformatics analysis [28]. Briefly, after trimming and quality control, reads were used for de novo assembly to build longer contigs from the non-redundant reads by using a CLC assembler (de novo assembly) with default options: word size 20, bubble size 50, simple contig sequences and min 35 nt length (Table S2). Annotation of these contigs was performed using the blastn algorithm with default options (thread 1, word size 11, match 2, mismatch 3, gap cost existence 5, extension 2) and the NCBI Plant-hosted Viral Reference genomes (downloaded on 13 May 2021). In parallel, the reads were directly mapped to the reference genome of AHVd, ALV-1 and both genomic RNAs of CCGaV, and were counted with and without redundancy (using the map to the reference command allowing 1 mismatch). The number of normalized reads (read/1 million reads—RPM) was calculated as the ratio of mapped redundant reads to the number (in millions) of total sequenced reads.

### 2.4. Virus Diagnostics by RT-PCR

RNA extracts were reverse-transcribed by a RevertAid First Strand cDNA Synthesis Kit (Thermo Fisher) using random primers according to the manufacturer’s instructions. Subsequent PCR analysis (Table S3) was performed with Phire Hot Start II DNA Polymerase (Thermo Fisher) or PPP Master Mix (Top-Bio, Vestec, Czech Republic). The quality of the cDNA was tested by amplifying a part of the *Malus domestica* actin gene. The PCR products were evaluated by gel electrophoresis. PCR products intended for Sanger sequencing were amplified with Q5 DNA polymerase (New England Biolabs, UK) and then cut and purified from the agarose gel using a GeneJET Gel Extraction Kit (Thermo Fisher). The products were Sanger-sequenced directly or cloned into the CloneJET vector (Thermo Fisher). Sequences of the PCR products or the cloned viruses were deposited in NCBI GenBank (Table S4).

### 2.5. RT-qPCR

RT-qPCR assays were conducted on a CFX96 real-time PCR detection system (Bio-Rad, Hercules, CA, USA). The 10 µL reaction was prepared from 5 µL of tenfold diluted cDNA, 0.25 µL of forward and reverse primers (10 mM, final concentration 250 nM, Table S3), 2.75 μL of nuclease-free water and 2 µL of 5× HOT FIREPol EvaGreen qPCR Mix Plus (Solis BioDyne, Tartu, Estonia).

The reaction conditions were set up with a three-step cycling protocol—95 °C for 12 min, followed by 40 cycles of 95 °C for 10 s, 60 °C for 20 s and 72 °C for 20 s. Dissociation curve analysis was performed by ramping from 65 °C to 95 °C (with increments of 0.5 °C for 5 s) to verify the specificity of primer amplification and the presence of potential primer dimers based on the presence of a single peak. No-template and positive controls were included to check for potential cross-contamination and presence of genomic DNA. NADH mRNA was used as an internal endogenous control for levels of plant material. (Table S3). The data were analysed using Bio-Rad CFX Maestro 1.1 (Bio-Rad) and R software version 4.1.0 [29] under RStudio version 2021.09.1+372.

### 2.6. Northern Blot Validation

For Northern blot analyses, 4–5 µg of total RNA was separated on formaldehyde-1.2% agarose gels and blotted to a Nytran NX membrane (GE Healthcare, Chicago, IL, USA) by the capillary method using 20xSSC. Hybridization was carried out at 65 °C in Church buffer (0.5 M phosphate buffer, pH 7.2 containing 1% BSA, 1 mM EDTA, 7% SDS) overnight with the appropriate radioactively labelled probe, washed for 5 min in 2 × SSC, 0.1% SDS and for 15 min in 0.5 × SSC, 0.1% SDS at the hybridization temperature and exposed to an X-ray film. AHVd specific, P32-labelled DNA probes were prepared by using the Thermo Scientific DecaLabel DNA labelling kit (Thermo Fisher). As a template, we used the PCR-amplified and purified product of a cloned viroid.

### 2.7. Biotest

Buds of ten tested trees were grafted to eleven indicators routinely used for apple biotests: Lord Lambourne, Spy227, R12740_7A, *Malus platycarpa*, Virginia crab, *Pyronia veitchii*, Stayman, Golden Delicious, Gravensteiner, Red Delicious, and *Cydonia oblonga* in August 2016. As CCGaV was previously known to infect only citrus, during this test, there were no known indicator species that could detect its presence. Symptoms on the indicators were evaluated in the upcoming 1st, 2nd and 3rd years. Leaf samples from the indicators used to test viral infection in the Florina cultivar were collected after two years in 2018. From them, RNA was isolated, and the presence of CCGaV was tested as described above. Leaves from the original Florina cultivar, sprouted on *C. oblonga* and *M. platycarpa*, were also collected and tested for the presence of the virus.

### 2.8. Origin of Trees for RT-PCR Survey

In Hungary, trees from five production orchards in the Nyirseg region were tested: two Florina orchards, two Akane orchards randomly (in 2020), and one orchard in Olcsvaapati (in 2017 and 2020) where scab-resistant Re-cultivars, namely, Renora, Remo, Relinda, Rebella and Reglindis, are grown (in 2017 and 2020) (Table S1B).

The origin of the Re-series trees dates back to the germplasm in the isolator house at Ujfeherto. We could test Renora, Remo, Relinda, and Rebella mother trees grown grafted on M9 rootstock, while from Reglindis, there was no available mother tree isolated. Virus-eliminated trees of the candidate cultivars (Rosmerta, Hesztia, Artemisz, Cordelia) were also grown in the same isolator but on their own roots. Trees in this isolator were tested in 2020. RT-PCR tests of the ten trees that were included in the pooled sRNA HTS test were repeated in 2018 and in 2019, but at this later point, all of the individuals of the same variety were tested to determine the infection frequency.

We also tested the collection of ancient Hungarian varieties at Erd (43 trees, in 2019) and a new collection at the same place (13 trees, in 2020). Altogether, we tested 102 trees from Hungary.

### 2.9. Grafting

Two HTS-tested varieties, Golden Delicious (*n* = 50) and Red Boskoop (*n* = 50), were grafted onto commercial certified M9 rootstocks grown in the spatially isolated nursery of the Research and Breeding Institute of Pomology (RBIP) following good phytosanitary practices in August 2020. A year later, scion leaf samples were collected and tested for the presence of ALV-1 and CCGaV.

### 2.10. Molecular Identification of Aphid Samples

Taxonomic identification of aphid species was performed using molecular barcoding of the *coxB* gene with published primers [30] and Phire Tissue Direct PCR Master Mix following the manufacturer’s recommendations (Thermo Fisher). The GenBank accession numbers are provided in Table S4.

### 2.11. Data Analysis

The sequence data were analysed using Geneious Prime^®^ 2022.1.1 (Biomatters, Auckland, New Zealand) and CLC Genomics Workbench 9.5.1 (Qiagen, Hilden, Germany). The RT-qPCR data were processed with Bio-Rad CFX Maestro 1.1, version 4.1 (Bio-Rad), and further data analyses were conducted in R version 4.1.0 (2021-05-08) [29] and the ggplot2 package version 3.3.5 [31] under RStudio version 2021.09.1+372.

## 3. Results

### 3.1. HTS Diagnostics

To investigate the virus status of apple orchards and apple germplasm collections, HTS was applied as an unbiased diagnostic method using dsRNA/total RNA or sRNA in the Czech Republic and Hungary, respectively (for details, see Appendix A). In total, 61 apple leaf samples from germplasm collections, private orchards, or rootstocks were processed by RNASeq of ds/total RNA in the Czech Republic (CZ) or by HTS of sRNA in Hungary (HU) (Table A1). Three particular HTS runs consisted of 6 or 10 individual samples pooled together, and the remaining leaf samples were sequenced by HTS individually. Due to the pooling strategy, individual samples were retested by means of either RT-PCR with target-specific primers or Northern blot (Table S3).

Only 5 out of 23 (21%) rootstock samples lacked any viral signatures, and the rest (79%) were infected by ALV-1, AHVd, CCGaV, and/or undescribed blunervirus. Similarly, 21 of 25 germplasm samples (84%) were infected with ALV-1, AHVd, ASGV, CCGaV, Solanum nigrum ilarvirus 1 (SnIV-1), the blunervirus, or their combinations. Notably, neither rootstock nor cultivar trees showed any virus-like symptoms.

Apart from one virus-free specimen, quite diverse viromes were discovered among the 13 samples from private orchards. These were taken from rather old apple trees and contained viruses not observed in rootstock or germplasm samples, including several recently described agents, such as cherry virus Trakiya (picornavirus) and Malus domestica virus A (velarivirus).

Presence of each detected virus in individual leaf samples (i.e., also in individual samples within pooled mixture) was confirmed either by specific RT-PCR with Sanger sequencing or by Northern blot. Furthermore, specific RT-PCR also confirmed the presence of ALV-1 in three additional germplasm samples previously diagnosed ALV-1-negative by HTS of sRNA (Appendix A).

### 3.2. RT-PCR Screening of AHVd, ALV-1 and CCGaV

**Figure 1:**
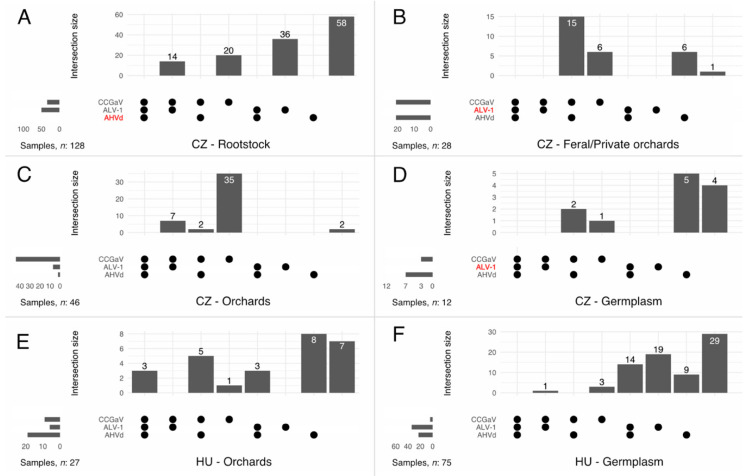
UpSet plots of apple hammerhead viroid (AHVd), apple luteovirus 1 (ALV-1), and citrus concave gum-associated virus (CCGaV) infections in the Czech (**A**–**D**) and Hungarian (**E**,**F**) samples uncovered by high-throughput sequencing and RT-PCR screening. Horizontal bars show the total number of viroid- or virus-positive cases. Vertical bars display the number of negative, single, and mixed infections. Pathogen names in red highlight the absence of positive cases. For example, panel A shows rootstock Czech samples, where AHVd was not detected. The total number of ALV-1 cases was higher than that of CCGaV cases (horizontal bars). Fourteen cases of mixed ALV-1/CCGaV infection were detected, while twenty and thirty-six samples were singly infected by CCGaV and ALV-1, respectively. Fifty-eight samples were tested as negatives.

As AHVd, ALV-1 and CCGaV were the most frequently detected agents by HTS, we designed an RT-PCR survey to obtain further insight into their distribution. We surveyed trees of different origins (germplasm collections, private orchards, production orchards and rootstock materials) from different locations in the Czech Republic (*n* = 214) and in Hungary (*n* = 102) and found infections in 149 (70%) and 45 (44%) cases, respectively (Figure 1, Table 1).

Of the three viruses tested, single infections dominated among infected trees (CZ—51% and HU—63%), followed by double (CZ—19%, HU—20%) and rare triple (HU—4%) infections (Figure 1). AHVd infections were abundant (70% of tested trees were viroid-positive) in the Hungarian production orchards analysed, and ALV-1 infections were more prevalent in the germplasm collections (45%), both in the open field and under the isolator, with 37% and 45% of virus-positive cases, respectively. In some cases, we tested the same trees in different years (the year of detection is indicated in Table S1).

AHVd was found in several places in CZ and HU, but its infection rate was twice as high in the open-field production orchards than in the germplasm collection, 70% and 31%, respectively.

The ALV-1 infection rate in the isolator house at Ujfeherto (HU) for the new cultivar candidates growing on their own root was 29%, while 63% of the Re-series cultivars grafted on M9 were found to be ALV-1 infected.

CCGaV infection was found in 14 cases (12%) among the Hungarian samples, affecting 8 cultivars: Golden, Jonagold, Reglindis, Rebella, Remo, Florina, Akane and Artemisz. Four out of five Reglindis trees were infected with CCGaV. For this cultivar, there is no mother tree under the isolator, and the stocks are prepared by grafting the existing tree at production orchards, which could be the reason for the high infection rate. Both Florina samples tested CCGaV-positive, including samples from the new collection. In the old collection, the Florina tree used for sRNA HTS was the only tree out of four that survived until the end of 2019, showing severe decline and trunk decay symptoms (Figure S3).

Only a minority of positive samples were infected with more than one pathogen (Figure 1). Interestingly, some sample types were negative for either AHVd (CZ-Rootstocks) or ALV-1 (CZ-Germplasm, CZ-Private orchards) (Table 1).

### 3.3. Phylogenetic and Sequence Variability Analyses

#### 3.3.1. AHVd

**Figure 2:**
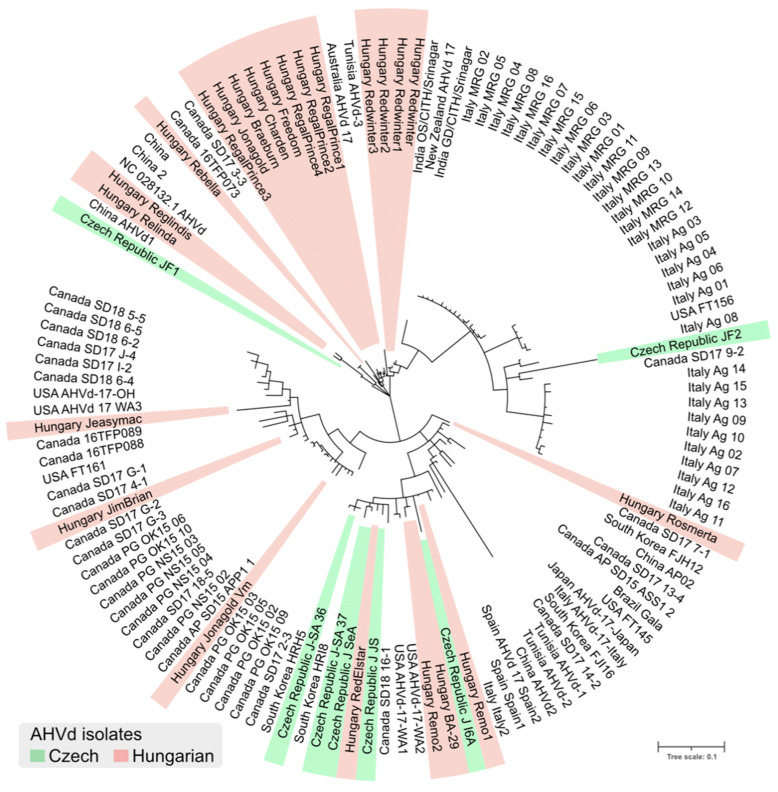
Phylogeny of AHVd isolates from the GenBank database and the current study. Czech and Hungarian isolates are highlighted. All branches with support less than 0.8 were collapsed.

We determined the full genomes of seven Czech and eleven Hungarian AHVd variants. Sequences of the variants showed 77–99% identity to each other (Table S5). According to the phylogenetic analysis, they cluster independently from their geographical origin (Figure 2).

**Figure 3:**
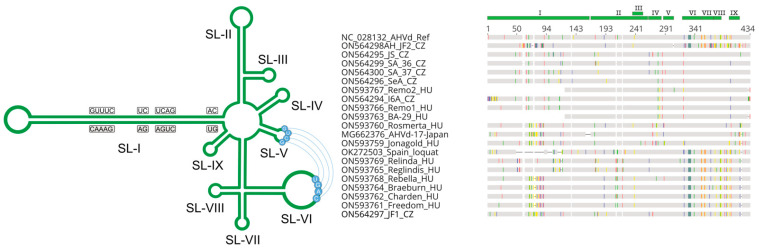
Molecular organization and alignment of AHVd variants, indicating the stem-loop structure (I-IX) above the sequence alignment and on the schematized AHVd structure. The structure cartoon was prepared using the verified secondary structure of AHVd [11].

AHVd variants were 433–434 nt long, with the exception of Remo2 and BA-29, which were much shorter. In these 308 nt long variants, a 120 nt deletion in the Stem-Loop I (SL-I), the hammerhead-motif-containing part of the viroid genome, is present. Detailed investigation of the viroid sequences showed very high similarity within the stem-loop parts of the viroid genome (Figure 3).

Variations were present almost exclusively in the loop part of the structure, where base pairing is not essential for the proper viroid function. Sequences of variants from the same cultivar showed slight differences and clustered together, indicating their common origin and further evolution in the infected trees.

**Figure 4:**
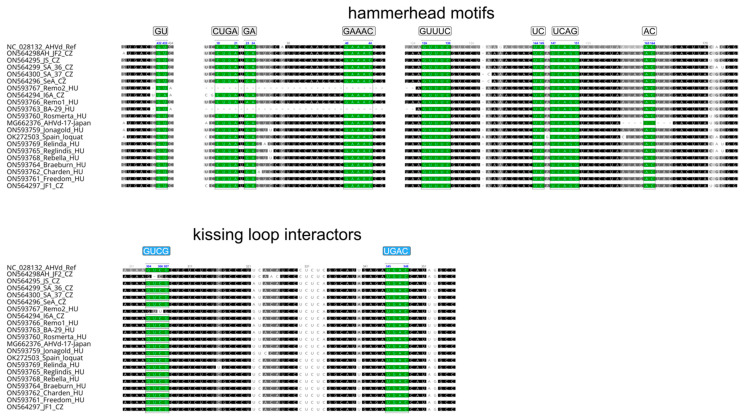
Alignment of the hammerhead-motif-containing part of the AHVd variants, including the reference-resembling and deletion-containing variants from Japan and the Spanish loquat. Residues forming the hammerhead motifs are highlighted in green.

A search for the hammerhead self-cleaving domain showed that it is present in all but the Remo2 and BA-29 variants (Figure 4).

Residues involved in the kissing-loop interaction are the same as in the Chinese reference genome (enabling four interactions), except for one Czech isolate (JF2) (Figure 4). In this variant, similar to AHVd-IT-MRG_01, a deletion is present at stem-loop (SL)-V, but because of a further variation in the interacting SL-VI, interaction in this putative kissing-loop domain is possible through three base pairs [15].

**Figure 5:**
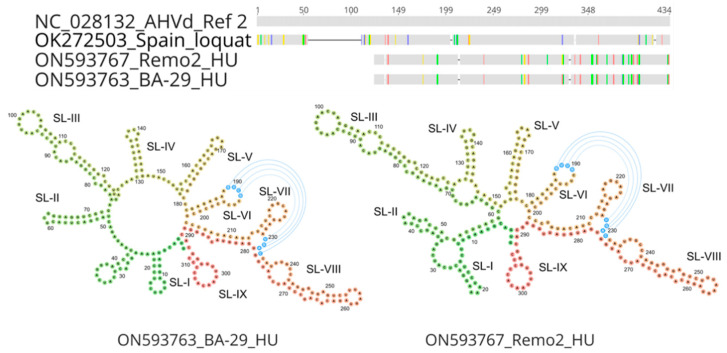
Alignment and secondary structure prediction of AHVd variants with deletions. The upper part shows the differences within their coding region, while the lower part shows the modelled secondary structures of the BA-29 and Remo2 AHVd isolates prepared using Vienna RNAfold software. The predicted stem-loops (SL-I to SL-IX) are indicated. Residues involved in the kissing-loop interaction are labelled by blue circles and lines.

The secondary structure of the Remo2 and BA-29 variants showed that except for the hammerhead stem-loop, they have a very definite secondary structure (Figure 5).

#### 3.3.2. ALV-1

**Figure 6:**
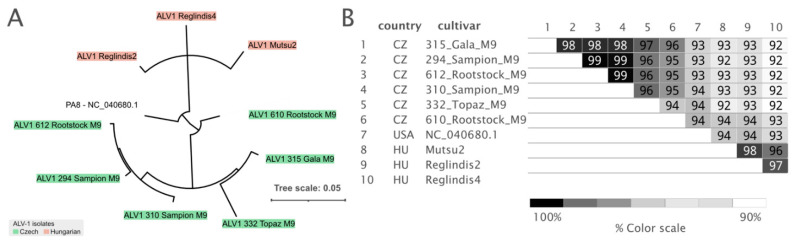
(**A**) Phylogeny of nearly complete genomes of ALV-1. All branches with support less than 0.8 were collapsed. (**B**) Nucleotide identity between the ALV-1 genomes.

Nine nearly complete ALV-1 genomes were reconstructed. Sequence identity and phylogenetic analyses divided them into two groups (Figure 6), while the PA8 isolate was left as an outgroup. All Hungarian and Czech ALV-1 isolates clustered based on country. Notably, isolates M9-610 and M9-612, originating from the same batch of rootstock material, were separated on different tree branches (Figure 6A). The estimated nucleotide variability reached up to 8% (Figure 6B). Most changes were found in the ORF1 and ORF5 regions (Figure S4).

**Figure 7:**
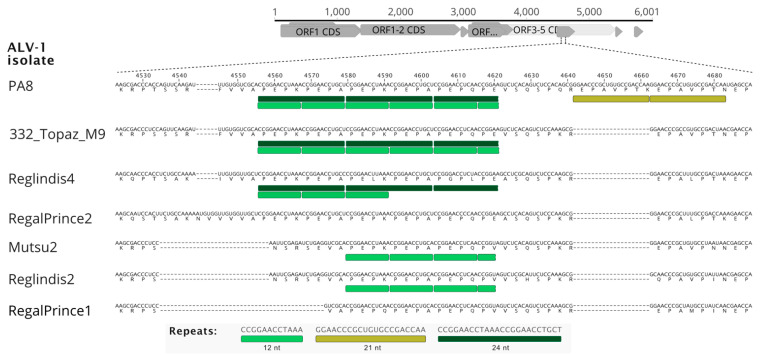
Multiple nucleotide alignment of the ORF5 region of the ALV-1 isolates with identified tandem repeats. The PA8 isolate was used as the reference. Deduced amino acid sequences are shown under respective nucleotide triplets. Gaps are shown as dashes. Consensus sequences of three kinds of tandem repeats are shown under the alignment.

Multiple nucleotide alignment of six ALV-1 sequences revealed a genomic region (~4500–~4700 nt) with several insertion/deletion events of variable length (Figure 7 and Figure S4).

Interestingly, all of the indels were in-frame, without coding frame disruption of the P3-P5-encoded protein. The BLAST-based search of the insertion sequences against the GenBank database did not reveal any significant hits (Accessed on 20 May 2022; E-value cut-off 0.5). As there were noticeable repeats consisting of the PEPK/A motif in the translated sequence, we analysed all available ALV-1 sequences for the presence of tandem repeats (TRs). Repeats of three different lengths were found—12 nt, 21 nt, and 24 nt. Although the algorithm used identified 12 nt and 24 nt repeats as separate (Table S7), the 24 nt-long unit consisted of two 12 nt repeats (Figure 7; however, this unit was not completely identical within isolates (detailed alignment is shown in Figure S5). A further peculiarity is that the repeats were not found in all isolates. Remarkably, the PA8 isolate of ALV-1 had a unique two-copy repeat in the 4640–4682 region (Figure 7). While there were differences between the presence and number of TRs between isolates, the other source of nucleotide differences was several insertion/deletion events (Figure 7). While one indel was associated with the 21 nt repeat in the PA8 genome (Figure 7), the remaining TRs had no direct influence on the indels.

From a genomic organization point of view, ALV-1 is a unique luteovirus encoding two additional putative proteins: P1a (P0) and P5a. Putative ORF0 was predicted in all obtained ALV-1 isolates within the ORF1 borders. While the abovementioned indels and TRs did not influence P3-P5-encoded protein expression, ORF5a (P5a) was truncated up to 93% of its length in several isolates (Figure S4).

#### 3.3.3. CCGaV

**Figure 8:**
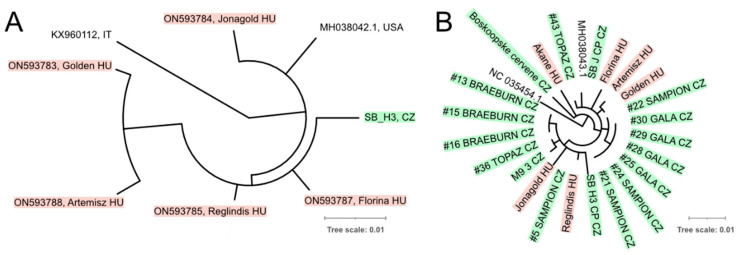
Phylogenetic inference of CCGaV isolates based on portions of RNA1 (**A**) and RNA2 (**B**). Czech and Hungarian isolates are highlighted. All branches with support less than 0.8 were collapsed.

Isolates of CCGaV infecting apple were grouped separately from the citrus isolates (Figure 8). No country-based segregation was observed. The shared nt identity between the apple isolates of CCGaV exceeded 98% (Figure S6).

### 3.4. Biotest of the CCGaV-Infected Florina Cultivar

Trees from the germplasm collection at Erd representing 10 cultivars were grafted onto indicator species for routine biotesting. Symptoms on indicators were evaluated after 1, 2 and 3 years. Among the ten cultivars, Florina was the only one that we found to be CCGaV-infected and that showed signs of trunk disease (Figure S3). Although there were no symptoms on the indicators used to test this cultivar, we wanted to check whether the virus could infect them. The 11 grafted indicators were tested after three years (Figure S7). CCGaV could only be detected in the Lord Lambourne indicator, while all the others tested negative. The original Florina sprouted on *M. platycarpa* and *C. oblonga*, and CCGaV could be detected there, suggesting that CCGaV failed to move from the budwood to the indicator and that the lack of the virus in the budwood was not responsible for the negative virus test, at least for these two indicators.

### 3.5. Graft Transmission of ALV-1- and/or CCGaV from the Virus-Positive Rootstock Material

**Figure 9:**
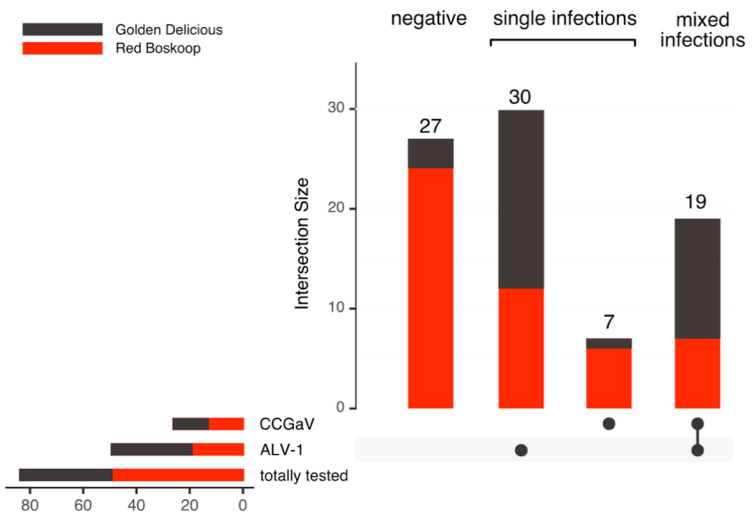
UpSet plot of infections in Red Boskoop (RB) and Golden Delicious (GD) scions grafted on M9 rootstocks. Horizontal bars show the total number of virus-positive cases. Vertical bars display the number of negative, single, and mixed infections.

One hundred commercially purchased M9 rootstock accessions were used for budwood grafting (August 2020) of two HTS-tested virus- and viroid-free apple cultivars (Red Boskoop (RB), *n* = 50 and Golden Delicious (GD), *n* = 50) with the purpose of establishing a virus- and viroid-free orchard. Nevertheless, we found that some of the M9 rootstocks were infected with either ALV-1, CCGaV, or both agents (Table S1A-rootstocks). Thus, a year later (September 2021), scion leaves were sampled and screened for virus presence. Out of 100 grafted trees, 17 declined (16 GD and 1 RB), and 56 out of the remaining 83 scions were found to be virus-positive (Figure 9, Table S8).

**Figure 10:**
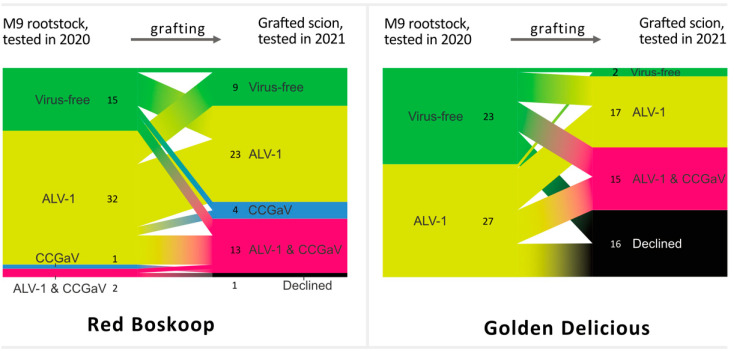
Details on graft transmission of ALV-1 and CCGaV from the M9 rootstock to RB and GD apple. The ALV-1/CCGaV status of the declined samples was not determined.

A majority of infections were ALV-1 cases (49), which were almost twice the number of CCGaV cases (26), with the latter predominantly found in the mixed mode with ALV-1. However, a number of these ALV-1- and/or CCGaV-positive scions were grafted onto previously virus-negative rootstocks (Figure 10), suggesting the existence of some vector(s) transmitting ALV-1 and CCGaV. This is further supported by many cases where a particular virus was not transmitted by grafting onto virus-positive rootstock (e.g., virus-free scions after grafting onto ALV-1-positive rootstock, ALV-1- and CCGaV-infected scions grafted onto ALV-1-positive or even virus-free rootstock).

In the case of the GD variety, the virome of 16 trees could not be determined as the trees declined before testing.

### 3.6. Potential ALV-1 Vectors

Thirteen pooled aphid samples were prepared from aphid colonies collected on apple trees. Ten adult animals were pooled for each sample. Molecular barcoding classified five samples as *Aphis pomi* (apple aphid, GenBank accession OP060740) and the remaining as *Dysaphis plantaginea* (rosy apple aphid, GenBank accession OP060741).

All aphids were tested negative for AHVd, and one aphid sample (1879) was found to be positive for CCGaV. In this case, nevertheless, Ct values for CCGaV were comparable to values for the NADH mRNA apple internal control in the sample (Table S9). This finding suggests CCGaV detection from the diet source. In contrast, four rosy apple aphid pools were positive for ALV-1 while being negative for apple internal controls.

## 4. Discussion

In recent years, high-throughput sequencing (HTS) has not only led to the discovery of new viruses [8,32] but also uncovered the complexity of metaviromes in field-collected samples [33]. This also includes viruses replicating in plant-associated organisms, which are inevitably present in field-collected samples. Nevertheless, some of these were later found to replicate in the plants [34].

The aim of our study was not to compare the results from the Czech Republic and Hungary but to mutually complement and discuss the data obtained in parallel studies by both teams, who met during an international conference. During a virus survey in apple trees, we found a widespread distribution of AHVd, ALV-1 and CCGaV both in the Czech Republic and in Hungary, where they have not been reported previously. Both ALV-1 and CCGaV were present in different kinds of apple trees—germplasm and rootstock materials—as well as in production and private orchards. In contrast, we have never detected AHVd in rootstock trees, indicating that there is another route of its introduction and/or spread. A moderate to high incidence of AHVd was documented in germplasm collections, which might be a potential source for its further propagation. As the incidence of AHVd was higher in orchards, factors other than propagation factors could be involved in AHVd dissemination.

A high molecular AHVd variability within the isolates was caused by both single nucleotide differences and deletion events, which have also been described in previous studies [13,17,35]. Due to this variability, AHVd isolates clustered into several groups without any country-based segregation. We also detected AHVd genomes with a 120-nt deletion in the SL-I structure (isolates Remo2 and BA-29), which possesses conservative residues within the hammerhead self-cleaving domain. In the Remo2 isolate, a full-length variant (Remo1, 433 nt) without the deletion was found in the same tree. It is possible that the Remo2 variant is a defective form that co-replicates with the full-length wild-type Remo1. In the case of BA-29, the deletion was exactly at the same position; however, we did not find any other variant in the tree. Therefore, the origin and consequence of this deletion on the functionality of the viroid are still elusive. The shortest AHVd genome described thus far was an isolate from loquat from Spain (AHVd-SL73.6) comprising 376 nt, which is a result of a 56-nt-long deletion in SL-I [17]. It is not known, however, whether the tree was infected with any other sequence variants of AHVd. Other deletion events near functionally important hammerhead self-cleaving sites were found in AHVd-17-Japan (9 nt), SD17_13-4 (7 nt), and AHVd_17_Spain2 (24 nt) [13,35]. Nevertheless, their hammerhead self-cleaving motif was not disrupted and can fold in a similar structure in silico as the reference genome.

In contrast to AHVd, ALV-1 was detected in rootstock at a high incidence. ALV-1 has been discovered in declining trees infected with other common apple viruses. Although rapid apple decline (RAD) was suggested to be linked to the presence of ALV-1, there are certainly also cases of ALV-1 infection without RAD. Nevertheless, RAD likely has an asymptomatic period followed by the development of trunk cankers and cracks [18]. In our study, a number of ALV-1-positive apple trees originated from orchards aged 5 to 15 years. Visual estimation of trees did not reveal any cases of frequent declines in tree rows; however, RAD development might still be caused by specific virus strain(s) on sensitive varieties. The original ALV-1 study reported a high incidence of RAD coinciding with the M9 rootstock, suggesting M9 sensitivity to ALV-1 as the primary cause of disease development [18]. While in Korea 30% of the tested M9 rootstocks (*n* = 20) were found to be ALV-1 infected [19], supporting this hypothesis, in Greece its presence could be not correlated to M9 or rootstock origin [20].

ALV-1 was widespread both in the production orchards and in the germplasm collection. Its origin is elusive and it cannot be exclusively connected to the use of the M9 rootstock there. For example, virus-positive samples were found among the trees grafted on MM106 but also among the trees from the other germplasm collections grafted on M4 and M6. However, it is true that the highest incidence of ALV-1 was observed in cultivars grafted on M9 roots or in M9 rootstock material (Table 1). Testing the latter revealed the additional presence of CCGaV. These rootstocks originated from three separate purchases, and several different isolates of ALV-1 were found infecting the largest tested rootstock batch. Thus, we assume that the rootstocks were obtained from individual mother trees that may be the source of the ALV-1 heterogeneity.

Single nucleotide changes and indel events contributed to the observed variability in the ALV-1 isolates. The first described isolate, ALV-1, was predicted to contain two additional ORFs absent in other luteoviruses, ORF0 and ORF5a, with possible protein-coding activity [18]; however, the existence of these proteins has not yet been verified experimentally. Interestingly, polemoviruses, poleroviruses and enamoviruses encode viral suppressors of RNA silencing (VSRs) at the 5′ end of their genomes, and the start codons of these ORF0-encoded proteins are shifted to the 5′ termini, while ORF0 of ALV-1 overlaps completely with ORF1 (Figure S-ALV-1-GenALN). Moreover, other known luteoviruses do not have any ORFs at this position, and their P4 protein was found to show VSR activity [36]. The presence of this putative P0, which is conserved in all of the sequenced variants, raises the possibility that it also acts as a VSR, but this question should be investigated in the future.

Interestingly, the identified indels within the ALV-1 genomes affected the predicted ORF5a by the introduction of termination codons at different positions. While these indels did not have any influence on the P3-P5 protein coding capacity, their presence led to truncation of the predicted ORF5a; therefore, its existence and essence for proper viral function can be questioned.

Although CCGaV was widespread in the tested trees in the Czech Republic, in Hungary, it was found less frequently than either AHVd and ALV-1. CCGaV was shown to be the causative agent of a disease in citrus, but there are no described CCGaV-specific symptoms in apple, and we also could not connect its presence to any specific symptoms. An exception was the CCGaV-positive Florina tree manifesting severe decline and trunk decay. As the Florina and Akane germplasm collections were infected with CCGaV, we further surveyed two Akane and two Florina orchards in Nyirseg. As these were CCGaV-negative, we assume that the origin of the infection is somewhere in the germplasm collection rather than in the open field.

CCGaV isolates showed much less sequence variation within their sequenced region. Apple isolates formed a separate cluster from the citrus CCGaV isolate, and their variability was less than 2%. Successful CCGaV transmission to the Lord Lambourne indicator and to the RB and GD cultivars indicates a potential mechanism of CCGaV introduction into orchards. It is not clear if failure of CCGaV to infect the other indicators is systemic or is related to a specific combination of CCGaV isolate and incompatible cultivar.

Grafting of two virus-free cultivars on M9 rootstock with various virus statuses and subsequent virus testing of scions a year after the procedure revealed an increased incidence of ALV-1, CCGaV, or both. As viral heterogeneity in the rootstocks used (despite having been purchased as a single batch) can explain some of these cases, there is likely a secondary route of virus transmission via an unidentified vector. Numerous aphids are known as established vectors for poleroviruses [37], and whiteflies have been proven to be vectors of a recently discovered polerovirus infecting pepper [38]. In our study, while some rosy apple aphids tested ALV-1-positive, all apple aphids returned negative. As there are cases when virus-positive aphids failed to introduce a polerovirus into the plant [38,39], rosy apple aphids stand as a prospective ALV-1 vector candidate and should be further experimentally tested. Interestingly, all tested aphid samples were CCGaV-negative, and although fungi-mediated transmission was suggested, there are still no known CCGaV vectors. However, the existence of vector transmission was further supported by the fact that both ALV-1 and CCGaV were detected in previously virus-free scions grafted onto virus-free rootstocks, and there were also cases when viruses were apparently not transmitted from infected rootstock to scions. The alternative explanation for the increase in virus-positive cases is that the viral concentration and/or distribution in the rootstock tree is not uniform and may be below the detection limit of the assay used. However, three or four leaves were sampled for each analysis, which should address potentially uneven viral distribution.

The remaining question is the impact of newly discovered viruses on plant health status. The established orchard with RB and GD cultivars infected by either ALV-1, CCGaV or both will serve as the experimental model in the following years.

## 5. Conclusions

Our study provides new data and a substantial extension of knowledge in the following areas:

1. Occurrence and high incidence of AHVd, ALV-1, and CCGaV in germplasm, mother stock, propagation materials, orchards and feral trees in CZ and HU.
2. Insight into the variability of these pathogens by HTS and Sanger sequencing.
3. First data on the possible spread of ALV-1 and CCGaV by rootstock, grafting, and possible vectors and hence a need for testing of apple propagation material for ALV-1 and CCGaV to limit their spread into new orchards.

We believe that our extensive data on AHVd, ALV-1, and CCGaV in apple, the principal fruit crop in Europe, will be of interest to a broad community of apple breeders, nurseries, apple growers and phytosanitary administrations.

## Figures and Tables

**Table 1:** Percentage of virus- and viroid-positive samples divided by categories (by country, sample type, and rootstock).

Country	Type Sample	Rootstock	Percentage Positive, %			Total Trees, *n*
			AHVd	ALV-1	CCGaV	
CZ	Germplasm	no	58	0	25	12
	Feral/Private orchards	unknown	75	0	75	28
	Production orchards	M9	4	15	96	46
	Rootstock material	M9	0	39	27	128
HU	Germplasm	M4	35	56	5	43
		M9	0	63	0	8
		MM106	23	15	8	13
		no	29	29	14	7
		unknown	75	25	0	4
	Production orchards	MM106	81	38	44	16
		unknown	55	0	18	11

## Data Availability

The Fastq files of sRNA HTS are accessible at GEO, series accession number GSE205183. The sequences of the virus variants are available at NCBI GenBank with the following accession numbers: ON854958–ON564300 (CZ) and ON593759–ON593794 (HU).

## Supplementary Materials

The following supporting information can be downloaded at https://www.mdpi.com/article/10.3390/v14112347/s1, Figure S1: Geographical locations of the origin of the Czech (CZ) and Hungarian (HU) samples; Figure S2: Northern blot validation of the sRNA HTS; Figure S3: Photos of the Florina cultivar at Erd (Hungary) infected with CCGaV; Figure S4: Multiple nucleotide alignment of the ALV-1 isolates; Figure S5: Multiple nucleotide alignment of the ORF5 region of the ALV-1 isolates with identified tandem repeats; Figure S6: Shared nucleotide identity in a 1193-nt-long part of the polymerase gene between CCGaV isolates; Figure S7: RT-PCR test of the indicators used for biotesting the Florina cultivar for the presence of CCGaV RNA1 and RNA2; Table S1: Detailed information about the virus diagnostic survey in the Czech Republic (A) and in Hungary (B); Table S2: Initial statistics of the sequenced small RNA libraries; Table S3: Sequences of the PCR primers used for virus detection with their appropriate reference; Table S4: List of accession numbers produced and used in the study; Table S5: Nucleotide identity of AHVd isolates; Table S6: Detailed results of the bioinformatics analysis of small RNA HTS; Table S7: Results of identified tandem repeats in the sequences of ALV-1 isolates by Tandem Repeats Finder v. 4.0; Table S8: Test of leaves from M9 rootstock trees collected before grafting (2020) and scion leaves from 2021 (one year after grafting); Table S9: Cq results of RT-qPCR testing of pooled apple-associated aphid samples.

## Appendix A. Overview of Apple Samples Subjected to HTS

### Appendix A.1. Virus Diagnostics Using RNASeq

Thirty-six samples of three types (germplasm, private orchards, and rootstock) were subjected to RNA-seq analysis (Table A1). The pooling strategy was applied only for two batches of the M9 rootstocks, each with six individual samples. These rootstocks were found to be infected with AHVd, ALV-1, CCGaV, and a not-yet-described virus sharing molecular similarity with members of the *Blunervirus* genus (Table A1). Only 5 out of 23 rootstock samples lacked any viral signatures. Five out of six cultivar samples from the germplasm collection were infected with either AHVd, CCGaV, SnIV-1 or a blunervirus or by their combinations (Table A1). Notably, neither rootstock nor cultivar trees showed any virus-like symptoms.

In contrast, quite diverse viromes were discovered among the samples from private orchards, which were taken from rather old apple trees. However, one specimen was analysed as virus-free. In other samples, common apple viruses, which were absent both in rootstock and cultivar samples, were identified. In addition, several newly described agents were found there, such as cherry virus Trakiya, a picornavirus, and Malus domestica virus A, a velarivirus (Table A1). The presence of all viruses was verified by RT-PCR and Sanger sequencing.

### Appendix A.2. Virus Diagnostics Using HTS of Small RNAs

Samples were collected from five orchards and three germplasm collections at distinct geographical locations in Hungary (Figure S1). In total, 16 sRNA sequencing libraries were prepared (Table A1), originating from six locations, representing 18 different cultivars. The 8_Apple_biotest library was prepared by pooling RNA originating from 10 different cultivars at the same germplasm collection. A BLASTN search of the contigs revealed the presence of AHVd, ALV-1 and CCGaV. The RT-PCR or Northern blot investigation supported the HTS results (Figure S2). In one sample, we found apple rubbery wood virus 2, a virus that has not been described in Hungary before, and its presence should be validated in the future.

AHVd was found in 9 out of the 16 libraries. Validation by Northern blot was successful in all cases, including Red Elstar, Red Winter, Regal Prince and Jersey Mac, in the 8_Apple_biotest library (Figure S2). RT-PCR validation detected its presence in two additional samples, Jim Brian and Top Spur (Figure S2).

ALV-1 was detected only in the 8_Apple_biotest library by sRNA HTS and validated in eight out of ten cultivars, suggesting its widespread distribution at that location (Table A1). Although sRNA HTS did not predict its presence in any of the other 15 libraries, we could amplify the ALV-1-specific product in 10_Hesztia_of and 15_Artemisz_i, 16_Cordelia_i (Table A1).

The presence of both RNA1 and RNA2 of CCGaV was detected in five libraries by sRNA HTS: 5_Golden, 6_Jonagold, 7_Freedom, 8_Apple_biotest, and 15_Artemisz_i (Table A1). Its presence could be validated in four cases with our usual diagnostic primers but only with additional combinations (47F and 1514R for RNA1 and 23F and 1196R for RNA2) in the case of the 7_Freedom library. Testing individuals of the pooled germplasm samples showed that only the Florina cultivar was infected with this virus.

**Table A1:** Summary of HTS-tested samples.

Country, HTS Type	HTS Library Type of Sample *	Sample(s), *n*	HTS-Detected Viruses	
CZ, RNAseq	M9 1-6 R	M9 pooled, 6 samples	AHVd, CCGaV, blunervirus	
	M9 7-12 R	M9 pooled, 6 samples	AHVd, CCGaV, blunervirus	
	Golden Delicious 7B G	1	-	
	Šampion A G	1	AHVd	
	Jonagored Supra A G	1	AHVd, CCGaV, blunervirus	
	Red Boskoop A G	1	AHVd	
	Selena A G	1	CCGaV	
	Idared 6A G	1	AHVd, SnIV-1	
	JF_2_T P	1	AHVd	
	JF_1_PT P	1		
	J1-JIH 53 P	1	ACLSV, ASPV, MdoVA	
	M9-10 15 R	1	CCGaV, blunervirus	
	M9-27-E 83 R	1	-	
	M9-31-E 87 R	1	-	
	M9-3 19 R	1	blunervirus	
	Sampion 21 G	1	CCGaV	
	M9-10 R	1	CCGaV	
	M9-2 R	1	-	
	21-M9-5 R	1	-	
	130 P	1	CCGaV, ASGV, ASPV	
	131 P	1	ACLSV, AGCaV, ApLV, ASPV, CTV	
	723-M9 R	1	ALV-1	
	616-M9 R	1	ALV-1	
	610-M9 R	1	ALV-1	
	719-M9 R	1	-	
	644-H3-J P	1	CCGaV, ACLSV, AGCaV, ASGV, ASPV, ApLV, SnIV-1	
HU, small RNA	1_ZSz P	1	ACLSV, AMV, ASGV	
	2_SZH P	1	ASGV	
	3_SS P	1	-	
	4_Idared P	1	AHVd, ASGV	
	5_Golden P	1	CCGaV	
	6_JonagoldP	1	AHVd, CCGaV, AMV	
	7_Freedom P	1	AHVd, CCGaV, ACLSV, ASGV, ARWV-2 **	
	8_Apple_biotest G	pool of 10 different cultivars	AHVd, ALV-1, CCGaV	
	9_Rosmerta_of G	1	AHVd, ASGV	
	10_Hesztia_of G	1	AHVd, ASGV	
	11_Artemisz_of G	1	ASGV	
	12_Cordelia_of G	1	AHVd, ASGV	
	13_Rosmerta_I G	1	-	
	14_Hesztia_I G	1	AHVd ACLSV, ASGV	
	15_Artemisz_I G	1	CCGaV, ACLSV, ASGV	
	16_Cordelia_I G	1	AHVd	

Asterisk (*) marks sample types: G—germplasm, P—private orchard, R—rootstock. AGCaV—apple green crinkle-associated virus, AHVd—apple hammerhead viroid, ALV-1—apple luteovirus 1, ACLSV—apple chlorotic leafspot virus, AMV—apple mosaic virus, ApLV—apricot latent virus, ARWV-1—apple rubbery wood virus 1, ARWV-2—apple rubbery wood virus 2, ASGV—apple stem grooving virus, CCGaV—citrus concave-gum associated virus, CTV—cherry virus Trakiya, SnIV-1—Solanum nigrum ilarvirus 1. Two asterisks (**) mark virus that was not subjected to validation.
