# Supplementary material for: Detection of Apple Hammerhead Viroid, Apple Luteovirus 1 and Citrus Concave Gum-Associated Virus in Apple Propagation Materials and Orchards in the Czech Republic and Hungary
Source: Viruses. 2022 Oct 25;14(11):2347. doi: 10.3390/v14112347 (PMC9695845; doi:10.3390/v14112347)
Supplement: Supplementary file 1 [file viruses-14-02347-s001.zip › viruses-1983772-suppl-proofs.pdf]

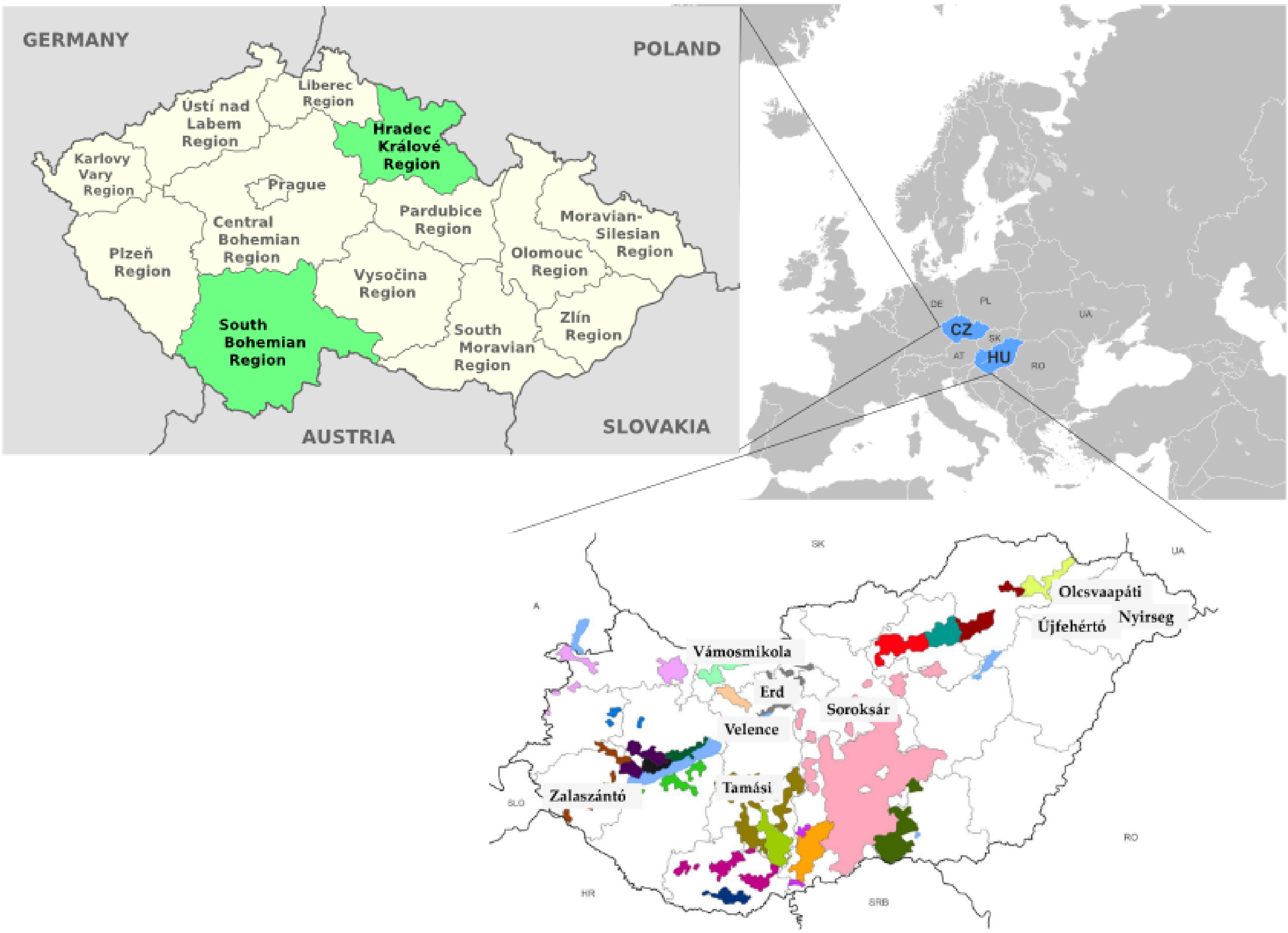

Figure S1. Geographical locations of the origin of the Czech (CZ) and Hungarian (HU) samples. The Czech samples were collected from private orchards in the South Bohemian Region. Samples from productive orchards and germplasm collection were taken in the Hradec Králové Region.

Productive orchards were sampled at Zalaszanto, Tamasi, Soroksar, Vamosmikola, Ujfeherto, Olcsvaapáti, and four more locations at Nyirseg, while certified stock collections are located at Erd, Soroksar and Ujfeherto (isolator houses are present only at Ujfeherto). Biotest were carried out at Velence on a Virology Station of NEBIH.

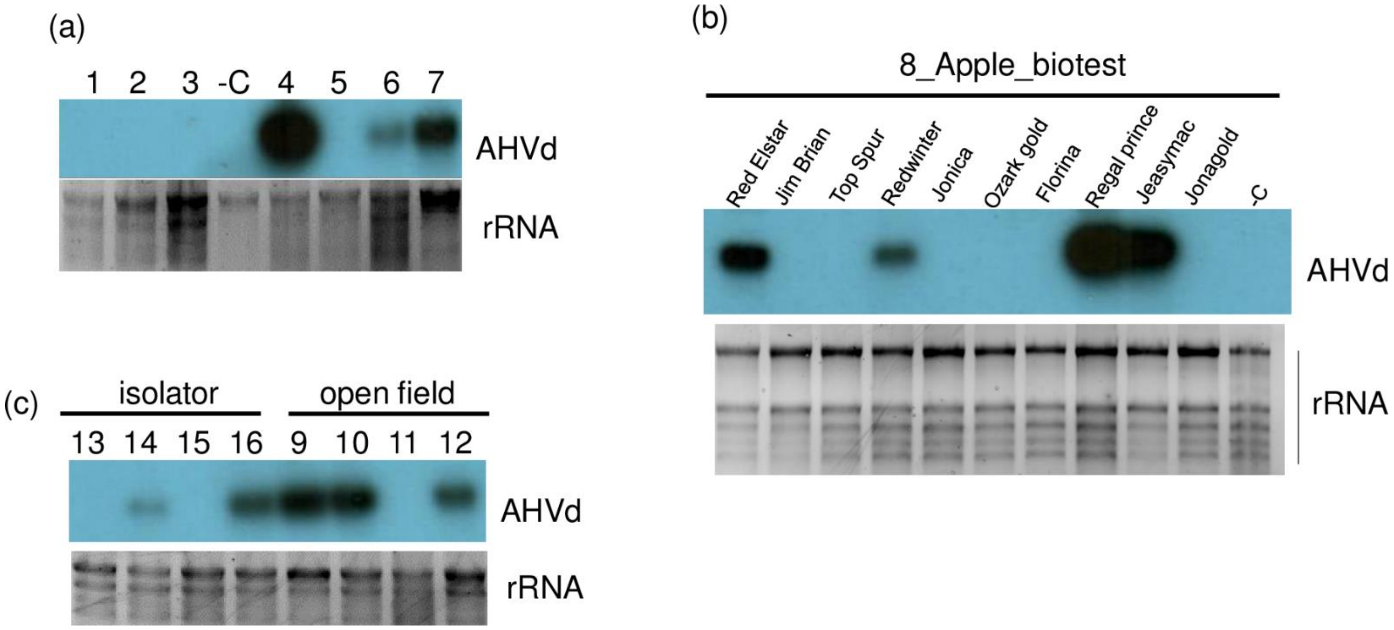

Figure S2. Northern blot validation of the sRNA HTS for presence of AHVd using AHVd specific radiolabelled probe (a) in libraries 1-7, (b) in individuals in 8_Apple_biotest library) and (c) in libraries 13-16. -C is a negative control.

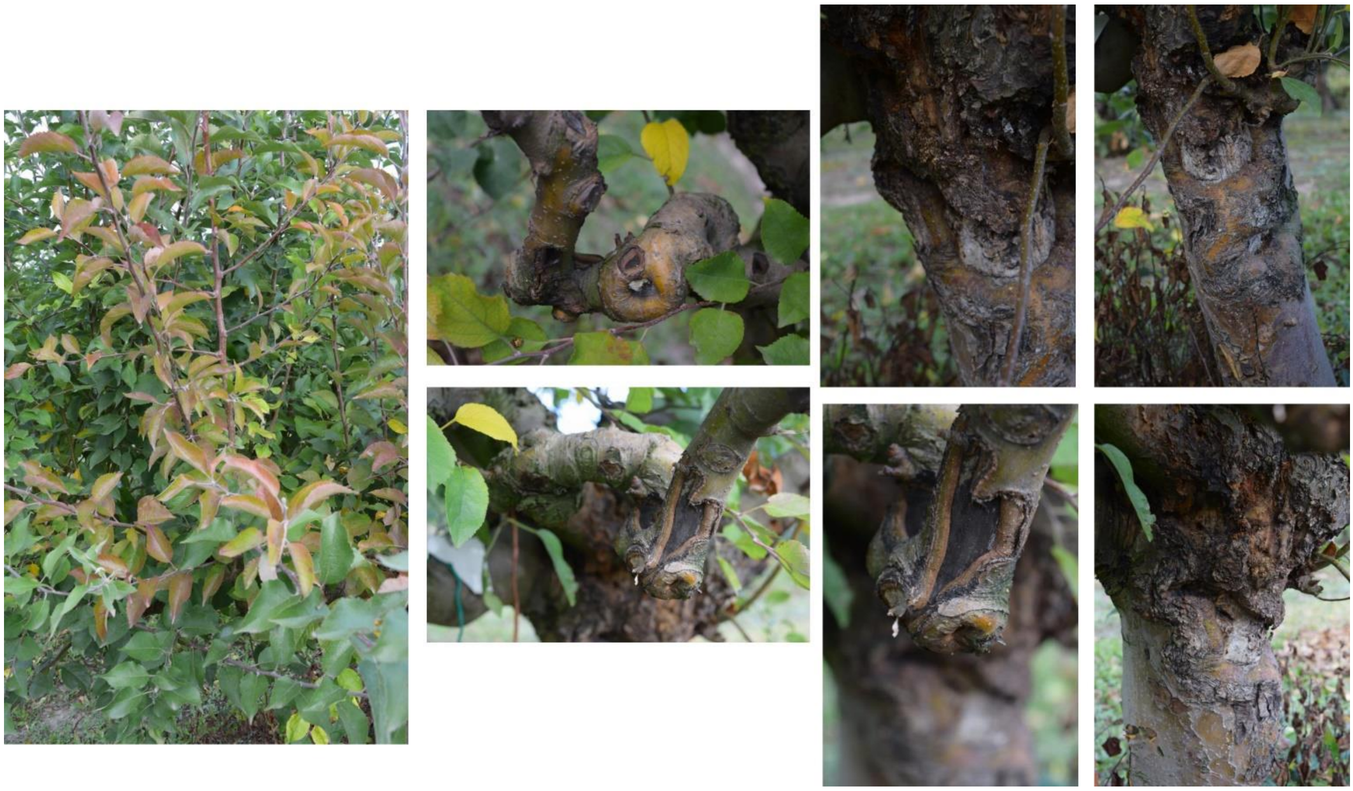

Figure S3. Photo of the Florina cultivar at Erd (Hungary), infected with CCGaV.

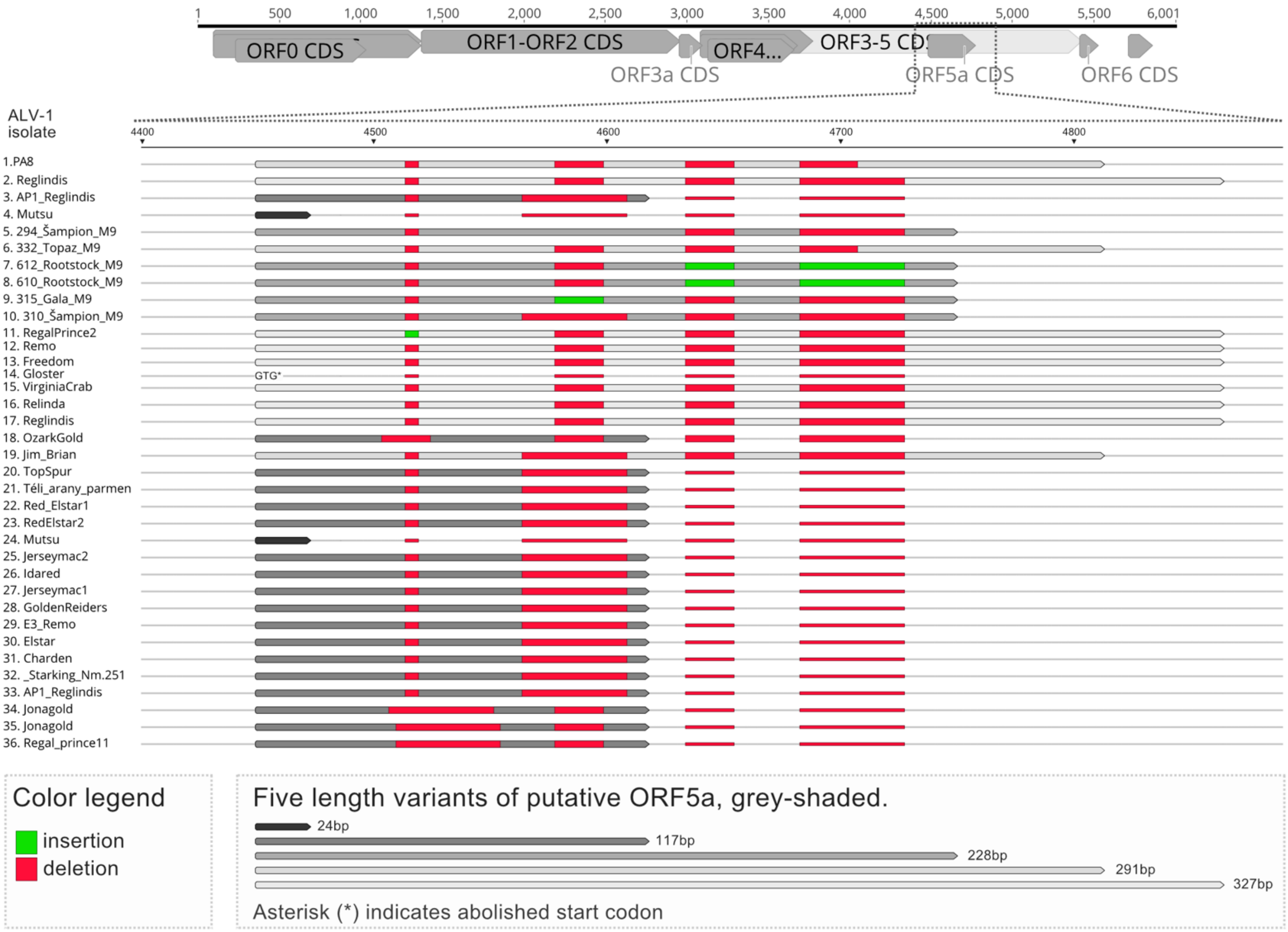

Figure S4. Multiple nucleotide alignment of the ALV-1 isolates. The PA8 isolate is used as the reference. Putative INDEL events are annotated as green/red boxes. There were five variants of the putative ORF5a that differed in length (24 nt – 327 nt). In addition, the Gloster isolate of ALV-1 had abolished start codon, annotated with an asterisk.

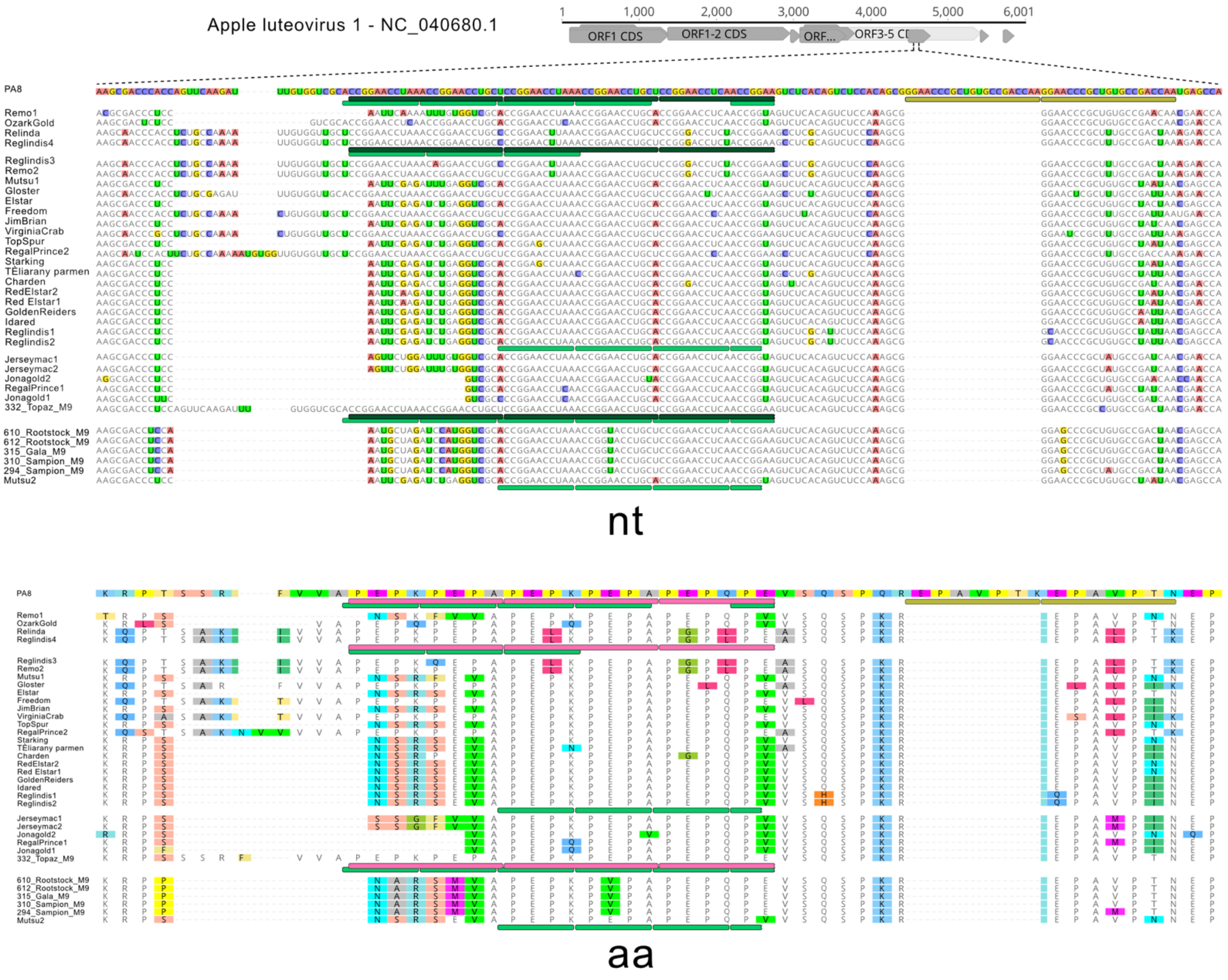

Figure S5. Multiple nucleotide alignment of the ORF5 region of the ALV-1 isolates with identified tandem repeats. The PA8 isolate is used as the reference. Deduced aminoacid sequences are shown in a lower part of the figure. Gaps are shown as dashes.

		1	2	3	4	5	6	7	8
1	ON593788, Artemisz HU		99	98	98	98	98	98	96
2	ON593783, Golden HU			98	99	98	98	98	96
3	ON593787, Florina HU				99	99	99	99	96
4	ON593785, Reglindis HU					99	99	99	96
5	ON593784, Jonagold HU						99	99	96
6	CCGaV; M9 644, CZ							99	96
7	MH038042.1, AC1, USA								96
8	KX960112, CGW2, IT								

Colour scale

100 99 98 97 96 95 94

Figure S6. Shared nucleotide identity in a 1193 nt long part of polymerase gene between CCGaV isolates. The Italian isolate CGW2 had a citrus host, while others have been isolated from apple hosts.

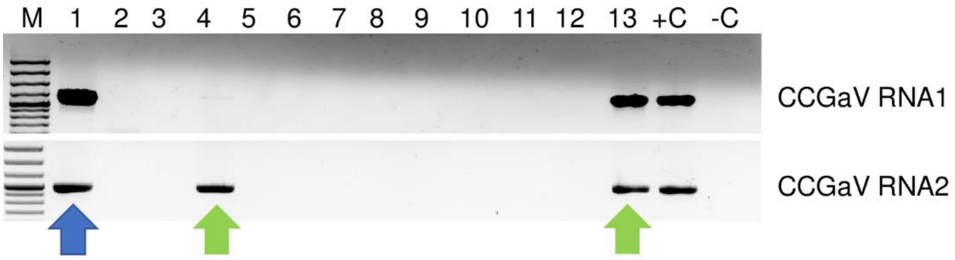

1	Lord Lambourne
2	Spy227
3	R12740_7A
4	Florina on M. platycarpa
5	Malus platycarpa
6	Virginia crab
7	Pyronia weitchii
8	Stayman
9	G. Delicious
10	Gravensteiner
11	Red delicious
12	Cydonia oblonga
13	Florina on C. oblonga

Figure S7. RT-PCR test of the indicators used for biotest of Florina cultivar for the presence of CCGaV RNA1 and RNA2. Blue arrows show results in the indicators, green arrows show results for the original cultivar sprouted during the biotest.
